# Time to Amyloid Positivity and Preclinical Changes in Brain Metabolism, Atrophy, and Cognition: Evidence for Emerging Amyloid Pathology in Alzheimer's Disease

**DOI:** 10.3389/fnins.2017.00281

**Published:** 2017-05-17

**Authors:** Philip S. Insel, Rik Ossenkoppele, Devon Gessert, William Jagust, Susan Landau, Oskar Hansson, Michael W. Weiner, Niklas Mattsson, for the Alzheimer's Disease Neuroimaging Initiative

**Affiliations:** ^1^Clinical Memory Research Unit, Department of Clinical Sciences Malmö, Lund UniversityMalmö, Sweden; ^2^Department of Veterans Affairs Medical Center, Center for Imaging of Neurodegenerative DiseasesSan Francisco, CA, United States; ^3^Department of Radiology and Biomedical Imaging, University of California, San FranciscoSan Francisco, CA, United States; ^4^Department of Neurology and Alzheimercenter, Neuroscience Campus Amsterdam, VU University Medical CenterAmsterdam, Netherlands; ^5^Alzheimer's Therapeutic Research Institute, University of Southern California, San DiegoSan Diego, CA, United States; ^6^Helen Wills Neuroscience Institute, University of California, BerkeleyBerkeley, CA, United States; ^7^Life Sciences Division, Lawrence Berkeley National Laboratory, BerkeleyBerkeley, CA, United States; ^8^Memory Clinic, Skåne University HospitalMalmö, Sweden; ^9^Department of Neurology, Skåne University HospitalLund, Sweden

**Keywords:** Alzheimer's disease, β-amyloid, atrophy, metabolism, cognition, preclinical

## Abstract

**Background:** Aβ pathology is associated with longitudinal changes of brain metabolism, atrophy, and cognition, in cognitively healthy elders. However, Aβ information is usually measured cross-sectionally and dichotomized to classify subjects as Aβ-positive or Aβ-negative, making it difficult to evaluate when brain and cognitive changes occur with respect to emerging Aβ pathology. In this study, we use longitudinal Aβ information to combine the level and rate of change of Aβ to estimate the time to Aβ-positivity for each subject and test this temporal proximity to significant Aβ pathology for associations with brain structure, metabolism, and cognition.

**Methods:** In 89 cognitively healthy elders with up to 10 years of follow-up, we estimated the points at which rates of fluorodeoxyglucose (FDG) PET, MRI, and cognitive and functional decline begin to accelerate with respect to the time to Aβ-positivity. Points of initial acceleration in rates of decline were estimated using mixed-effects models with penalized regression splines.

**Results:** Acceleration of rates of FDG PET were observed to occur 20+ years before the conventional threshold for Aβ-positivity. Subtle signs of cognitive dysfunction were observed 10+ years before Aβ-positivity.

**Conclusions:** Aβ may have subtle associations with other hallmarks of Alzheimer's disease before Aβ biomarkers reach conventional thresholds for Aβ-positivity. Therefore, we propose that emerging Aβ pathology occurs many years before cognitively healthy elders reach the current threshold for Aβ positivity (preclinical AD). To allow prevention in the earliest disease stages, AD clinical trials may be designed to also include subjects with Aβ biomarkers in the sub-threshold range.

## Introduction

According to the amyloid cascade hypothesis (Hardy and Selkoe, 2002), the accumulation of β-amyloid (Aβ) pathology in the brain is a key initiating event in the pathogenesis of Alzheimer's disease (AD). Downstream, the accumulation of Aβ is thought to lead to accumulation and spread of tau pathology (Braak and Braak, 1991; Schöll et al., 2016), brain hypometabolism, brain atrophy, and cognitive and functional decline (Bourgeat et al., 2010; Chételat et al., 2010; Landau et al., 2012; Insel et al., 2016a). Cerebrospinal fluid (CSF) biomarkers and positron emission tomography (PET) with tracers sensitive to Aβ, have made it possible to study the development of AD-related brain changes in the brain of living humans (Klunk et al., 2004; Perrin et al., 2009). Such biomarker-guided studies have contributed to the understanding that AD has a long prodromal [patients with mild cognitive impairment (MCI) who are Aβ+] and preclinical (cognitively normal individuals who are Aβ+) stage, where the build-up of brain pathologies is paralleled by a gradual development of cognitive and functional impairment (Zetterberg and Mattsson, 2014).

However, it is not clear at what degree of Aβ pathology downstream AD-related changes start to occur. For example, is it necessary to have fully established Aβ pathology before any changes in metabolism can be detected, or can such changes occur much earlier, before conventional thresholds for Aβ+ are reached? Previous studies of patients with MCI have indeed shown that biomarkers of Aβ are related to other hallmarks of AD, including brain atrophy, hypometabolism, and cognitive decline, already in measurement ranges prior to established thresholds for Aβ+ (Insel et al., 2015, 2016a). However, a limitation of these previous studies is that they rely on cross-sectional Aβ information, which may make it difficult to detect the earliest associations between Aβ and other hallmarks of AD. Detecting subtle changes in cognitively normal individuals is additionally challenging even with longitudinal Aβ information (Jack et al., 2013). Jack et al. compared a group of cognitively normal individuals observed to cross the threshold to Aβ+ during follow-up, although no differences in atrophy, FDG PET or cognition were found when comparing the stable Aβ− and incident Aβ+ groups. In another longitudinal study with serial Aβ measurements taken on cognitively healthy individuals (Mattsson et al., 2014), subtle increases in atrophy rates were observed in Aβ− subjects with incident Aβ+ subjects compared to Aβ− subjects with stable Aβ− levels. Taken together, the studies discussed above suggest that there may be an early stage of abnormal Aβ-metabolism, when biomarker signs of Aβ-pathology start to appear and are related to other aspects of AD, even before they become Aβ+, i.e., prior to meeting the conventional criteria for preclinical AD (Sperling et al., 2011). We call this stage of disease “emerging Aβ-pathology.”

The overall goal of this study was to test the hypothesis that emerging Aβ-pathology is coupled to other AD-related changes on a continuum over many years prior to the time when subjects cross the threshold to Aβ+. Using serial Aβ biomarker measurements taken over several years in cognitively healthy controls, we estimated rates of change of Aβ to calculate a time to Aβ+ for each subject. These subject-specific estimates of the proximity to the threshold for Aβ+ were then tested for associations with the longitudinal “downstream changes” in AD (FDG PET, brain volumetrics, and cognition). The specific goals of this analysis were to (1) identify if emerging Aβ-pathology is associated with other hallmarks of AD in cognitively healthy individuals and (2) estimate an order for the acceleration of the rates of change of several AD hallmarks as a function of Aβ accumulation.

## Materials and methods

### Standard protocol approvals, registrations, and patient consents

This study was approved by the Institutional Review Boards of all of the participating institutions. Informed written consent was obtained from all participants at each site.

### Participants

Data were obtained from the Alzheimer's Disease Neuroimaging Initiative (ADNI) database (adni.loni.usc.edu, www.adni-info.org). All sites had IRB approval to study human subjects. The population in this study included ADNI-1 and ADNI-2 participants enrolled into the cognitively normal cohort at screening, had a minimum of three longitudinal measurements of either CSF biomarkers or ^18^F-florbetapir PET, and were followed longitudinally for at least one of either magnetic resonance imaging (MRI), ^18^F-fluorodeoxyglucose (FDG) PET, or neuropsychological exams.

### Cerebrospinal fluid biomarker concentrations

Cerebrospinal fluid (CSF) samples were collected at baseline by lumbar puncture. CSF Aβ42 was measured by an xMAP assay (INNOBIA AlzBio3, Ghent, Belgium, Fujirebio), as described previously (Olsson et al., 2005; Shaw et al., 2009).

### MRI acquisition and processing

Structural magnetic resonance imaging brain scans were acquired using 1.5 Tesla MRI for ADNI-1 subjects and 3.0 Tesla MRI for ADNI-2 subjects. We used a standardized protocol including T1-weighted MRI scans using a sagittal volumetric magnetization prepared rapid gradient echo (MP-RAGE) sequence (Jack et al., 2008). Detailed descriptions can be found at www.adni-info.org. Automated volume measures were performed with FreeSurfer. We used data on combined volumetric measures from different Freesurfer regions of interest (ROIs), including the cingulate gyrus (rostral anterior cingulate, caudal anterior cingulate, posterior cingulate, isthmus cingulate) and the temporal lobe (hippocampus, entorhinal cortex, parahippocampus, superior temporal, middle temporal, inferior temporal, banks of the superior temporal sulcus, fusiform, transverse temporal, temporal pole, amygdala), parietal lobe (superior parietal, inferior parietal, supramarginal, postcentral, precuneus), frontal lobe (superior frontal, rostral middle frontal, caudal middle frontal, pars opercularis, pars triangularis, pars orbitalis, lateral orbitofrontal, medial orbitofrontal, precentral, paracentral, frontal pole) and occipital lobe (lateral occipital, lingual, cuneus, pericalcarine; Fischl et al., 2002; Insel et al., 2016a). We also evaluated the medial temporal lobe (hippocampus, entorhinal cortex, parahippocampus, amygdala, banks of the superior temporal sulcus) and lateral temporal lobe (inferior, middle and superior temporal gyri, fusiform, temporal pole, transverse temporal gyrus), and the hippocampus alone. Details about FreeSurfer parcellation can be found at https://surfer.nmr.mgh.harvard.edu/fswiki/CorticalParcellation. Left and right hemisphere volumes were averaged for each subregion.

### FDG PET

Methods to acquire and process FDG PET images were described previously. Full details of procedures and the standardized protocol are described at http://adni.loni.usc.edu/methods/pet-analysis/pre-processing/ and at http://www.adni-info.org/Scientists/ADNIStudyProcedures.html. Meta-ROIs included in the analysis were the temporal, angular, and posterior cingulate gyri (Jagust et al., 2010; Landau et al., 2011).

### Florbetapir PET

Similarly, methods to acquire and process ADNI florbetapir PET image data were described previously (Landau et al., 2012). Full details of acquisition and analysis can be found at http://adni.loni.usc.edu/methods/.

### Cognitive and functional outcomes

Cognitive measures assessed included the Mini-Mental State Examination (MMSE), Alzheimer's Disease Assessment Scale-cognitive subscale, 13-item version (ADAS13), immediate and delayed memory recall from the Wechsler Memory Scale (iMemory, dMemory), immediate and delayed Rey Auditory Verbal Learning Test (iAVLT, dAVLT), Trail Making Test parts A and B, Category Fluency, Boston Naming Test, Clinical Dementia Rating Sum of Boxes (CDR-SB), and the Functional Assessment Questionnaire (FAQ; Reitan, 1958; Rey, 1958; Pfeffer et al., 1982; Rosen et al., 1984; Wechsler, 1987; Williams et al., 1989; Morris, 1993).

### Statistical analysis

The predictor of interest in this analysis was the estimated time-to-Aβ+ (T2Aβ+). To be included in this analysis, subjects needed a minimum of three longitudinal measurements of Aβ, within the same amyloid modality (either CSF or PET). Because T2Aβ+ was not observed, linear mixed-effects models were fit to the longitudinal amyloid data by regressing Aβ levels on time since baseline measure (of either CSF or PET) to estimate subject-specific intercepts and slopes to calculate T2Aβ+ = (threshold−intercept)/slope. Because subject-specific slopes are unlikely to remain constant over time, we used estimates of acceleration and deceleration of Aβ rates (Toledo et al., 2013) to adjust individual slopes based on the distance from the threshold for Aβ+. We then compared these adjusted slopes to unadjusted slopes in a sensitivity analysis. We used previously defined thresholds of CSF Aβ_42_ <192 ng/L (Shaw et al., 2009) and florbetapir-PET SUVR >1.1 (Landau et al., 2012) to establish Aβ-positivity.

The relationship between longitudinal cognitive or imaging responses and T2Aβ+ was modeled in two steps. First, subject-specific rates for each response were estimated using linear mixed-effects regression with a random intercept and slope. Longitudinal cognitive scores were regressed on time (years) since initial visit while adjusting for age, gender and education. Volumes were regressed on time since initial visit while adjusting for age, gender, scanner type and intracranial volume. FDG-PET rates were estimated similarly, adjusting for age and gender.

In the second step, subject-specific rates were regressed on T2Aβ+ using monotone penalized regression splines. Generalized cross-validation was used to tune the smoothing parameter (Wood, 1994).

Steps 1 and 2 were repeated in 500 bootstrap samples to estimate 95% confidence intervals for the association between T2Aβ+ and each response using the 2.5th and 97.5th percentiles.

Permutation tests were performed to estimate the statistical significance of the association between T2Aβ+ and each response. Responses were regressed on permuted values of T2Aβ+ in each bootstrap sample to obtain a null distribution of F-statistics. *P*-values were then calculated as the proportion of null F-statistics that were equal or greater than the observed F-statistic estimated using the true T2Aβ+ labels.

For responses with a significant association with T2Aβ+, points of initial acceleration in rate of decline with respect to T2Aβ+ were estimated. These points were taken to be the moment the curves dropped one standard error (SE) below the mean response at the longest times to Aβ+. Standard deviations and 95% confidence intervals were estimated from the 500 bootstrap samples.

Baseline associations between demographics and T2Aβ+ were assessed using Spearman correlation for age and education and the Wilcoxon rank-sum test for gender. Data were inspected for entry errors and implausible values. All analyses were done in R v3.1.1 (www.r-project.org).

## Results

### Cohort characteristics

Eighty-nine cognitively normal participants (59 Aβ− and 30 Aβ+ at baseline) were followed longitudinally for up to 10 years. Participants had a mean age of 76 (62 to 90 years), 39 (44%) were female, they had a mean of 16 years of education (6 to 20), and 19 (21%) were APOE ε4 carriers. The cognitive battery was administered a median of 9 times over 9 years. MRI scans were done a median of 5 times over 2 years and FDG-PET scans were done a median of 2 times over 2 years. Cognitive and MRI analyses included all 89 participants, while FDG PET analyses included 78.

**Figure 1:**
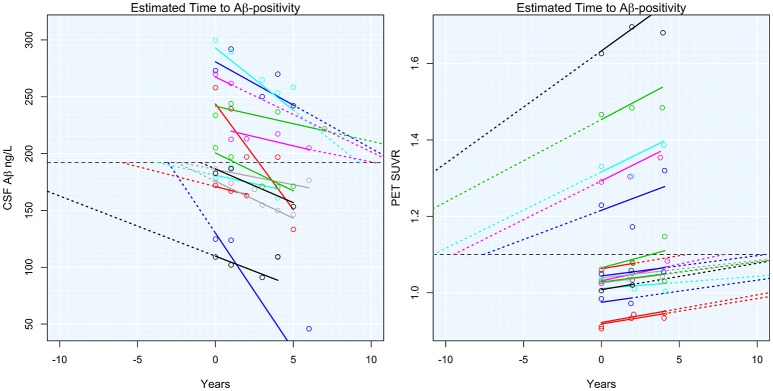
**Estimated time to Aβ-positivity**. Subject-specific estimated slopes of either CSF Aβ or PET Aβ are plotted against the time from baseline. Dashed colored lines show the projected time to or from Aβ-positivity. The horizontal dashed black lines indicate the threshold for Aβ-positivity.

Adjusted T2Aβ+ ranged from 52 to −27 years, with a negative number indicating time since becoming Aβ+. Subject-specific slopes and estimated T2Aβ+ are shown in Figure 1. T2Aβ+ was not associated with age (ρ = −0.05, *p* = 0.64), gender (*p* = 0.41), years of education (ρ = 0.12, *p* = 0.26) or Aβ modality (*p* = 0.38). On average, APOE-ε4+ individuals had 9 year shorter T2Aβ+ (mean = 0.8 years, *SD* = 17.7) compared with APOE-ε4− individuals (mean = 10.1 years, *SD* = 15.3, *p* = 0.01). During the 10 years of follow-up, 21 individuals progressed to a diagnosis of MCI and 11 individuals progressed to AD dementia. Progression was associated with shorter T2Aβ+ in AD progressors (mean = −6.27, *SD* = 14.7) compared with MCI progressors (mean = 6.10, *SD* = 17.5) or non-progressors (mean = 11.7, *SD* = 14.5, *p* = 0.001).

T2Aβ+ was not associated with increased odds of missing data over time in any of the analyses (*p* > 0.32).

### FDG PET

**Figure 2:**
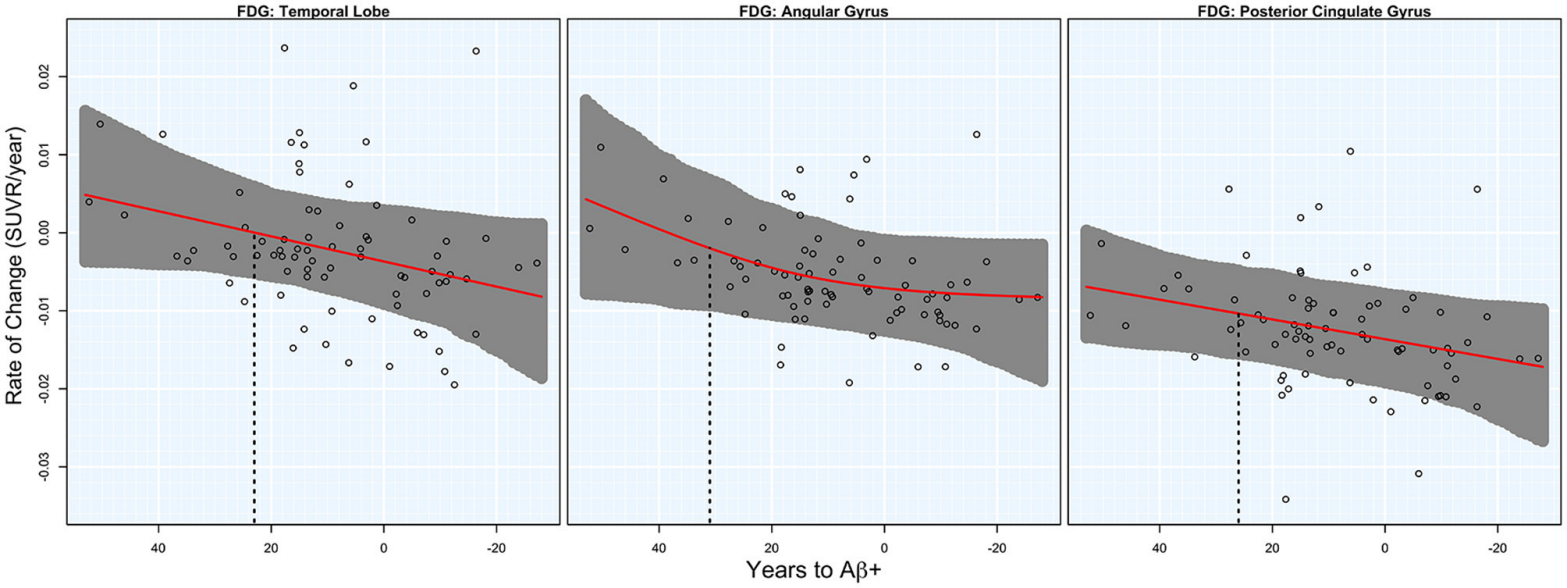
**FDG rates of change**. Annual rates of change for FDG PET are plotted against T2Aβ+. Rates worsen from top to bottom and time to Aβ-positivity decreases from left to right. Estimated curves are in red with 95% confidence intervals shaded in gray. The vertical dashed line in black is the estimate of the initial acceleration point.

Significant acceleration of rates of FDG PET was observed in all three ROIs. Acceleration was estimated to occur between 20 and 30 years before Aβ+. Estimates are summarized in Table 1 and plots are shown in Figure 2.

**Table 1:** **Estimates of initial acceleration points, 95% confidence intervals, and significance of permutations tests**.

**Outcome**	**Initial acceleration point and 95% CI (T2Aβ+)**	**Permutation test *p*-value**
**MRI**		
Temporal lobe	11 (33, −11)	0.044
MTL	–	0.066
LTL	–	0.094
Hippocampus	17 (42, −8)	0.036
Parietal lobe	–	0.360
Frontal lobe	–	0.390
Occipital lobe	–	0.646
Cingulate gyrus	–	0.054
**COGNITION/FUNCTION**		
ADAS13	5 (22, −12)	0.002
Category fluency	24 (53, −10)	0.002
iAVLT	16 (33, −1)	<0.001
MMSE	5 (35, −28)	0.006
Logical memory II	10 (42, −22)	0.02
FAQ	−2 (14, −18)	0.046
CDR-SB	2 (18, −14)	0.016
Trails A	–	0.062
Trails B	−3 (24, −28)	0.018
BNT	–	0.174
dAVLT	10 (42, −22)	0.010
**FDG PET**		
Temporal gyrus	23 (52, −6)	0.002
Angular gyrus	31 (53, 8)	<0.001
Cingulate gyrus	26 (48, 4)	0.010

### MRI

**Figure 3:**
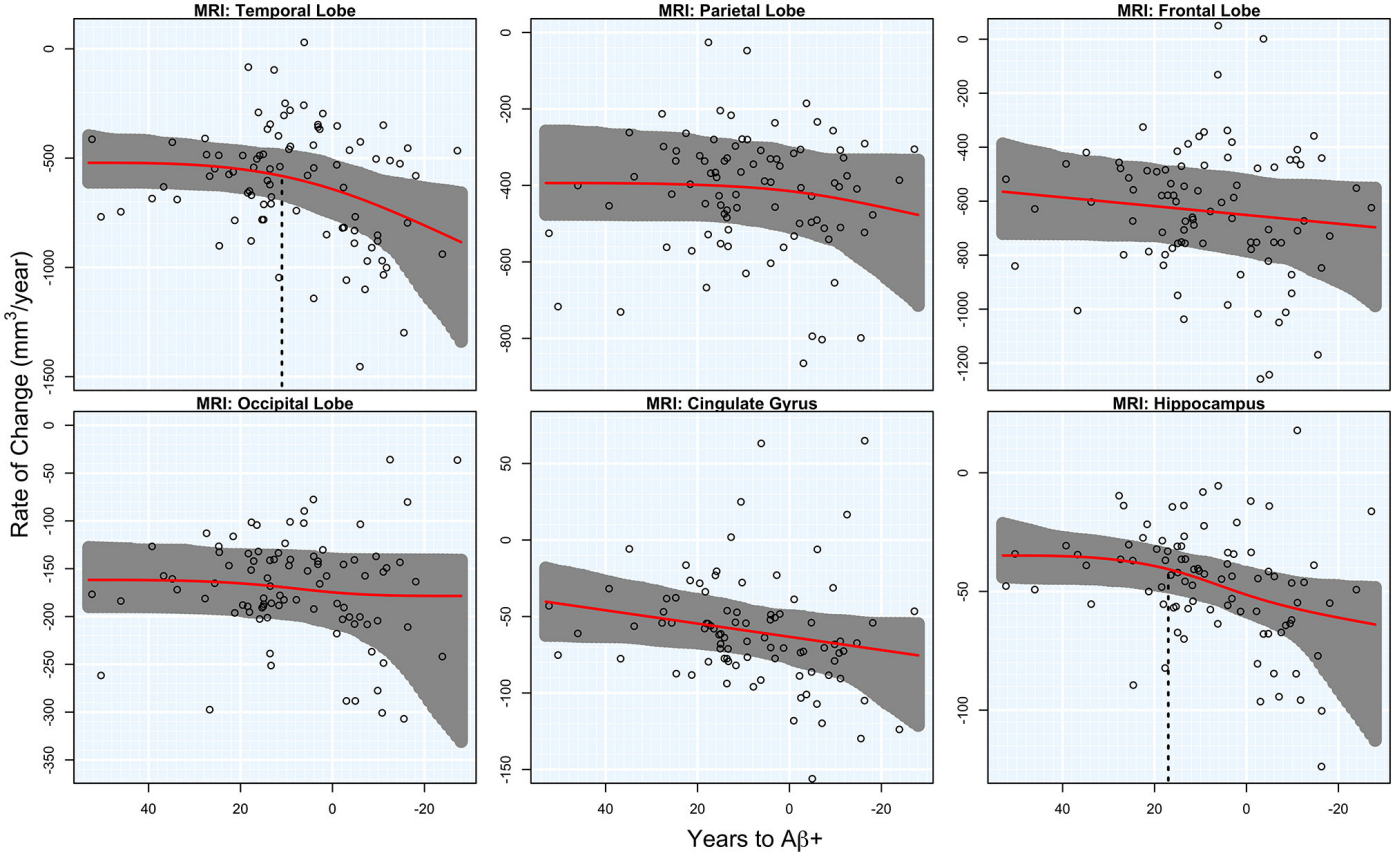
**MRI rates of change**. Annual rates of change of MRI regions are plotted against T2Aβ+. Atrophy rates increase from top to bottom and time to Aβ-positivity decreases from left to right. Estimated curves are in red with 95% confidence intervals shaded in gray. The vertical dashed line in black is the estimate of the initial acceleration point, if it exists.

There was significant acceleration of temporal lobe atrophy (*p* = 0.048), hippocampal atrophy (*p* = 0.036), and a trend in the cingulate gyrus (*p* = 0.054) with respect to T2Aβ+. The increase in hippocampal atrophy was estimated to start 17 years before Aβ+, while temporal lobe atrophy was estimated to start 11 years before Aβ+. We also examined the medial temporal lobe and the lateral temporal lobe separately. Acceleration in the MTL with respect to T2Aβ+ began 16 year before Aβ+ and 8 years before Aβ+ in the LTL, although neither region showed statistically significant acceleration (*p* = 0.066 and *p* = 0.094, respectively). No acceleration was found in the parietal, frontal or occipital lobes. Results of the overall tests of association between T2Aβ+ and MRI ROIs and also estimates of initial rate acceleration points and confidence intervals are summarized in Table 1. Rates of all ROIs are plotted against T2Aβ+, as well as estimates of points of the initial rate acceleration, in Figure 3.

### Cognitive and functional outcomes

**Figure 4:**
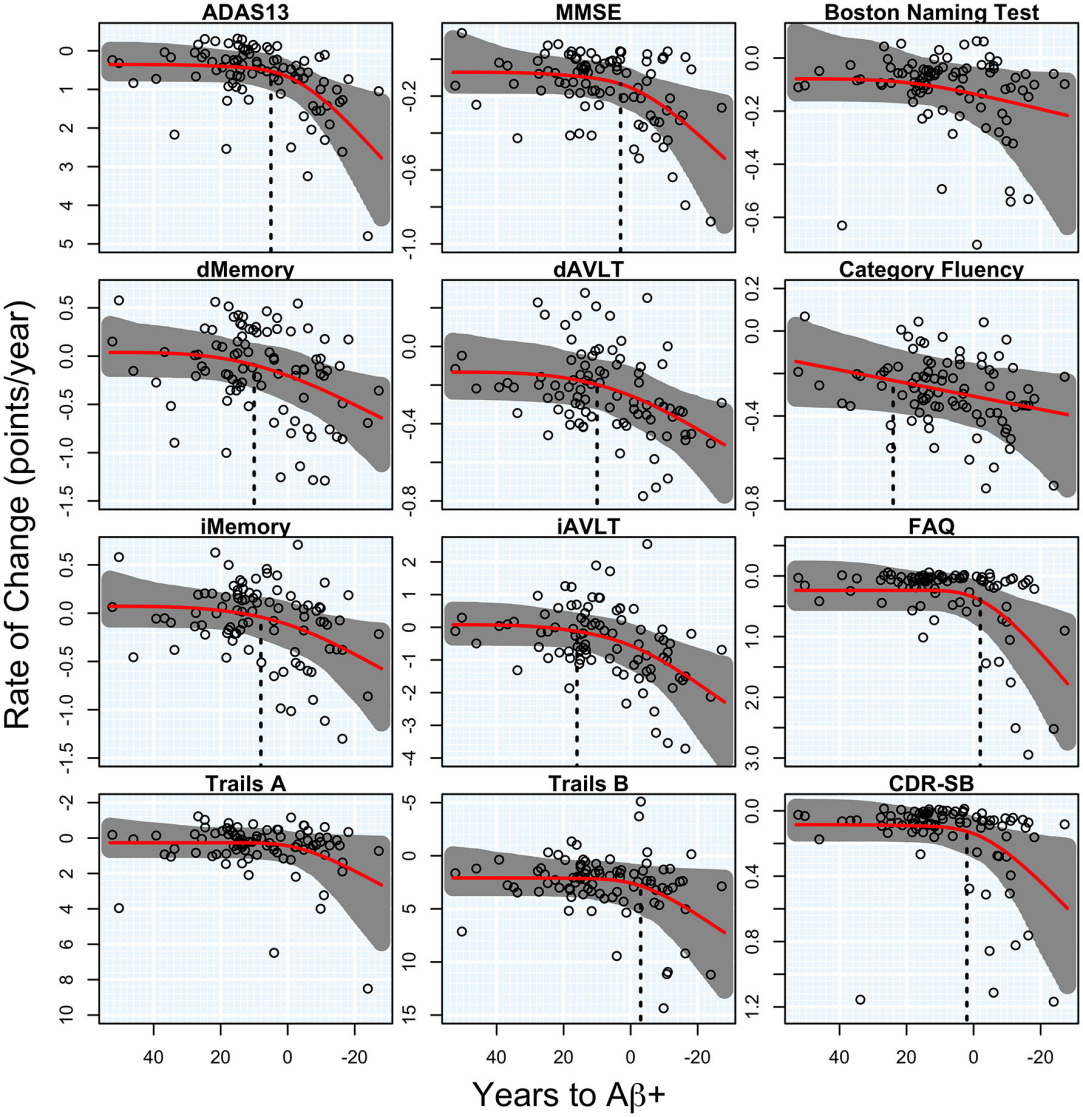
**Cognitive and functional rates of change**. Annual rates of change for cognitive and functional measures are plotted against time to Aβ-positivity. Rates of cognition or function worsen from top to bottom and time to Aβ-positivity decreases from left to right. Estimated curves are in red with 95% confidence intervals shaded in gray. The vertical dashed line in black is the estimate of the initial acceleration point, if it exists.

**Figure 5:**
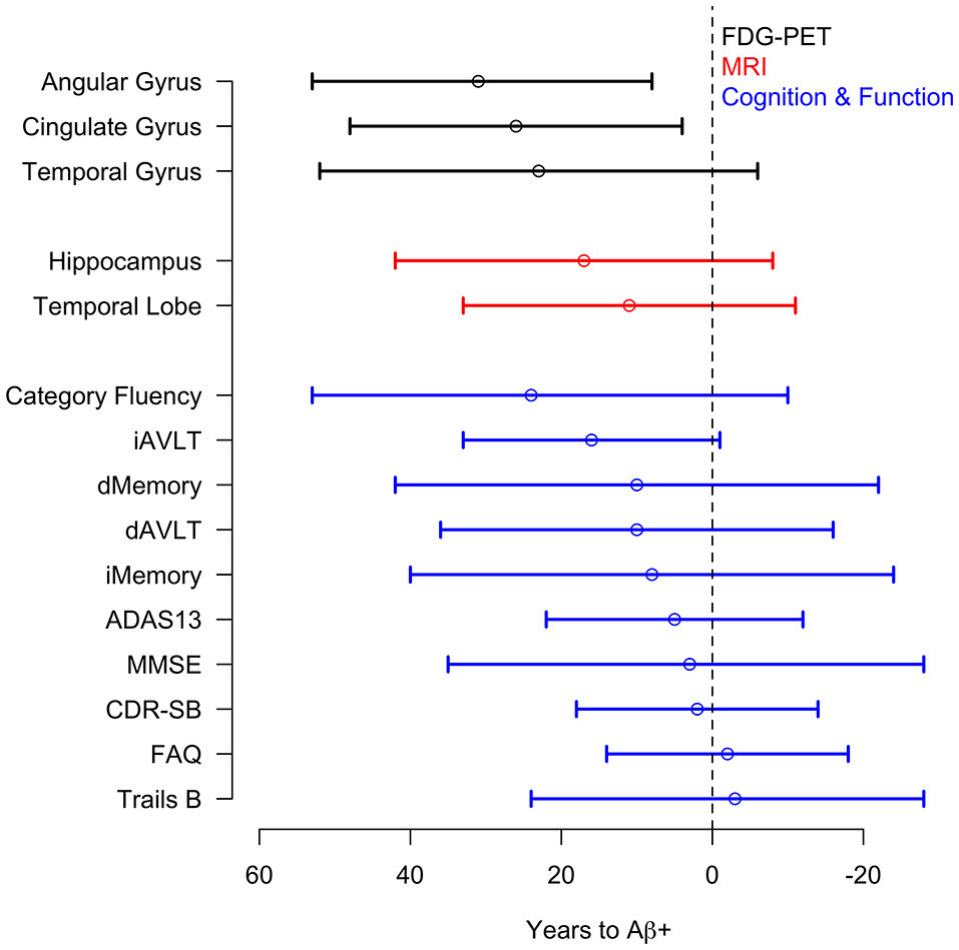
**Acceleration point estimates and 95% confidence intervals**. Estimates of the initial acceleration points and 95% confidence intervals for all outcomes are plotted against T2Aβ+. Time to Aβ-positivity decreases from left to right.

There was a significant acceleration in the rate of decline in 10 of the 12 cognitive and functional measures across the span of T2Aβ+. Decline in delayed memory recall was estimated to begin accelerating 10 years before Aβ+, with global measures of cognition following 5–8 years later. Hypothesis tests and estimates are summarized in Table 1. Plots of cognitive and functional outcomes are shown in Figure 4. All imaging and cognitive points of initial acceleration and confidence intervals are shown in Figure 5.

In a sensitivity analysis, we repeated all analyses without adjustments for the acceleration and deceleration associated with the distance from the threshold for Aβ-positivity. The acceleration point estimates were on average 5 years earlier compared with the adjusted estimates.

## Discussion

This study of longitudinal changes in Aβ biomarkers coupled with changes in brain metabolism, atrophy, cognition and function, showed that brain hypometabolism, hippocampal atrophy and subtle cognitive decline started to accelerate already very early during the development of Aβ pathology in cognitively healthy controls, before Aβ biomarkers reach conventional thresholds for Aβ+. Associations with changes in clinical function typically appeared later, around the time of Aβ+. Note that the changes in metabolism, volume and clinical signs were subtle, and at the early stage of disease that is studied here most subjects are still within normal levels of all studied variables.

The earliest changes associated with Aβ were in brain hypometabolism, which were seen in all tested regions: angular, temporal, and cingulate gyrus. These regions were selected based on their known associations with AD (Jagust et al., 2010; Landau et al., 2011). Reductions in metabolism were followed by changes in the hippocampus, language and immediate/delayed memory recall prior to Aβ+, while global cognition and function accelerated closer to the threshold for Aβ+. Acceleration of atrophy in the hippocampus and the entire temporal lobe occurred near the acceleration of memory recall, while atrophy in other lobes did not accelerate significantly during the study. The finding that T2Aβ+ was related to subtle changes in brain metabolism and cognition is in agreement with our previous findings from cross-sectional Aβ biomarker data in MCI subjects (Insel et al., 2016a). By incorporating longitudinal Aβ data and parameterizing the time variable as T2Aβ+, we showed, in cognitively healthy subjects, similar associations between early alterations in Aβ biomarkers and brain hypometabolism and cognition. The fact that most atrophy measures were not significantly accelerating before manifest Aβ+ was in line with previous results of atrophy only after brain hypometabolism and subtle cognitive changes appeared (Insel et al., 2016a). Our findings also suggested a spatial ordering of the associated regions for atrophy, since we could only detect acceleration of atrophy in the temporal, but not in the parietal or other lobes. The lack of atrophy in the parietal lobe is logical, since this brain region is typically only affected in clinical stages of AD, and may be associated with the spread of tau pathology to widespread cortical areas (Johnson et al., 2016; Schöll et al., 2016). Finally, the results suggest that some subjects may remain cognitively healthy several years after reaching the threshold for Aβ+. This is in accordance with previous reports showing that preclinical AD may persist for several years before development of significant cognitive impairment, i.e., prodromal AD (Vos et al., 2013; Jansen et al., 2015).

One important limitation of the analysis is that we did not have long enough follow-up to observe acceleration or deceleration of the Aβ biomarkers over time. We therefore attempted to adjust for hypothetical accelerations and decelerations. Results with and without adjustments for acceleration/deceleration of rates were similar in that both analyses estimated the acceleration of rates to occur prior to the threshold for Aβ-positivity. Although, the unadjusted analyses estimated acceleration to occur ~5 years earlier than the adjusted analyses. Given previous findings (Ossenkoppele et al., 2012; Villain et al., 2012; Villemagne et al., 2013), it is more likely that Aβ biomarkers accelerate as they approach Aβ+, and decelerate as Aβ+ is established, although the trajectories may differ slightly between CSF and PET Aβ biomarkers (Mattsson et al., 2015; Palmqvist et al., 2016). We therefore lean toward the estimates from the adjusted models and advocate caution when interpreting the exact T2Aβ+, especially near the tails of the distribution. Still, the results suggest that subtle changes in brain metabolism and cognition may develop several years prior to overt Aβ+. However, while there are clear associations between the T2Aβ+ and cognitive or biomarker changes, this does not imply a causal relationship between Aβ and these biomarkers. It is possible that the cause or causes of the early changes observed in FDG PET and cognition are associated with aspects of aging that are also associated with developing Aβ pathology, although all models are adjusted for chronological age. Previous studies have also shown that the *APOE*-e4 genotype has been linked to metabolic changes even in Aβ− people (Jagust and Landau, 2012).

Current clinical trials of novel disease-modifying AD drugs are mainly aimed at secondary prevention in people with established Aβ pathology, since they include subjects with either preclinical AD (Aβ+ cognitively healthy individuals), or prodromal AD (Aβ+ MCI), with Aβ+ defined using conventional thresholds (Sperling et al., 2014). The main finding of this study, that Aβ may be associated with changes in other hallmarks of AD before Aβ biomarkers reach conventional thresholds for AD, may have implications for the design of drug trials aimed at preclinical AD. To allow prevention in the earliest disease stages, studies may be designed to also include subjects with Aβ biomarkers in the sub-threshold range. This will introduce a problem of specificity since some subjects may have sub-threshold but stable Aβ pathology. One potential strategy to overcome this problem could be to create registries of volunteers with cross-sectional biomarker data who are followed longitudinally with cognitive tests. Individuals who decline in cognition over time may be at risk of developing Aβ pathology and could therefore be recruited to clinical trials for very early prevention (Insel et al., 2016b).

## Ethics statement

This study was carried out in accordance with the recommendations of the Institutional Review Boards of all of the participating institutions with written informed consent from all subjects. All subjects gave written informed consent in accordance with the Declaration of Helsinki. The protocol was approved by the Institutional Review Board.

## Author contributions

PI analyzed the data; PI and NM wrote the manuscript; PI, RO, DG, WJ, SL, OH, MW, and NM revised the manuscript.

### Conflict of interest statement

The authors declare that the research was conducted in the absence of any commercial or financial relationships that could be construed as a potential conflict of interest.
